# Tyrosine Kinase Inhibitors in pediatric chronic myeloid leukemia: a focused review of clinical trials

**DOI:** 10.3389/fonc.2023.1285346

**Published:** 2023-12-20

**Authors:** Fateen Ata, Maria Benkhadra, Rola Ghasoub, Liam J. Fernyhough, Nabil E. Omar, Abdulqadir J. Nashwan, Mahmood B. Aldapt, Kamran Mushtaq, Nancy A. Kassem, Mohamed A. Yassin

**Affiliations:** ^1^ Department of Endocrinology and Metabolism, Hamad General Hospital, Hamad Medical Corporation, Doha, Qatar; ^2^ Pharmacy Department, National Centre for Cancer Care and Research, Hamad Medical Corporation, Doha, Qatar; ^3^ Department of Medical Education, Weill Cornell Medicine Qatar, Doha, Qatar; ^4^ Health Sciences Program, Clinical and Population Health Research, College of Pharmacy, Qatar University, Doha, Qatar; ^5^ Department of Nursing, Hamad Medical Corporation, Doha, Qatar; ^6^ Department of Medicine, Unity Hospital/Rochester Regional Health, Rochester, NY, United States; ^7^ Department of Gastroenterology, University Hospital Southampton, Southampton, United Kingdom; ^8^ Department of Medical Oncology/Hematology, National Centre for Cancer Care and Research, Hamad Medical Corporation, Doha, Qatar

**Keywords:** Tyrosine Kinase Inhibitors, TKI, pediatric chronic myeloid leukemia, CML, Imatinib

## Abstract

Tyrosine Kinase Inhibitors (TKIs) is revolutionizing the management of pediatric Chronic Myeloid Leukemia (CML), offering alternatives to Allogeneic Hematopoietic Stem Cell Transplantation (AHSCT). We conducted a comprehensive review of 16 Randomized Controlled Trials (RCTs) encompassing 887 pediatric CML patients treated with TKIs including Imatinib, Dasatinib, and Nilotinib. The median patient age ranged from 6.5 to 14 years, with a median white blood cell count of 234 x 10^9/uL, median hemoglobin level of 9.05 g/dL, and median platelet count of 431.5 x 10^9/µL. Imatinib seems to be predominant first line TKI, with the most extensive safety and efficacy data. BCR::ABL response rates below 10% ranged from 60% to 78%, CCyR at 24 months ranged from 62% to 94%, and PFS showed variability from 56.8% to 100%, albeit with differing analysis timepoints. The Safety profile of TKIs was consistent with the known safety profile in adults. With the availability of three TKIs as first line options, multiple factors should be considered when selecting first line TKI, including drug formulation, administration, comorbidities, and financial issues. Careful monitoring of adverse events, especially in growing children, should be considered in long term follow-up clinical trials.

## Introduction

Chronic Myeloid Leukemia (CML) is a commonly occurring hematological neoplasm, in adults, caused by BCR::ABL1 gene mutation in the myeloid cells (1). Chromosome 22 (Philadelphia chromosome or Ph^1^) abnormality is seen in almost all patients (2). The median age of presentation is 50 – 60 years (3). Moreover, three clinically distinguishable phases can be seen in CML; chronic phase (CP), accelerated phase (AP), and blast phase (BP) (4). Extensive data exists for this age group concerning its molecular genetics, clinical course, and management (4). Although common in the adult population, CML is a very rare hematologic malignancy in the pediatric age group (< 2%), with knowledge gaps in the clinical behavior and management in this population group (5, 6).

Historically, allogenic stem cell transplant (SCT) has been the curative management for CML (7). Nevertheless, SCT can potentially present an array of acute and chronic severe complications, such as graft-versus-host disease, a range of cytopenias, mucositis, and persistent infectious conditions. (8, 9). In light of the significant adverse events associated with Stem Cell Transplantation (SCT), and subsequent to an extensive investigation into the genetic underpinnings of CML, the therapeutic approach to CML has witnessed a paradigm shift from invasive to non-invasive modalities. This shift, gravitating toward potentially less complex treatment strategies, has notably included the use of Tyrosine Kinase Inhibitors (TKIs) such as Imatinib. (10). TKIs inhibit the tyrosine kinase encoded by the ABL gene (1). Subsequent additions of newer TKIs such as nilotinib, dasatinib, and bosutinib have potentially made SCT a last resort in the management hierarchy, mainly reserved for CML – CP patients resistant to 2 TKIs and those with new mutations like T315I mutation (4, 11). Extensive research is now available on the use of TKI and the management of its adverse effects in adult population with CML (12–14).

TKIs work mainly by blocking the BCR::ABL pathway, resulting in the termination of cell proliferation without causing cell breakdown, causing around a 98 percent reduction in the abnormal myeloid cells, whereas leaving the normal cells undamaged (15). Imatinib, dasatinib, nilotinib, ponatinib, and bosutinib are currently used for CML in adults (6). Primary (failure to achieve optimum response) and secondary (relapse after optimum response) resistance with TKIs in CML-CP is seen in 25 and 15 percent of cases, respectively (16, 17).

While pediatric and adult CML may exhibit similar disease patterns and share certain genetic and phenotypic characteristics, variations are observed when evaluating the genetic and clinical phenotypes across these demographic cohorts. These variations span from microscopic disparities, such as the differential distribution of mutational breakpoints within the BCR::ABL1 oncogene, to macroscopic differences manifested in distinct prognostic outcomes and disease severity (18, 19). In pediatric CML, particularly during the blast phase, there is often a more pronounced lymphoid phenotype than the adult CML. This is accompanied by distinct cytogenetic aberrations that differ from those typically observed in the adult form of the disease. Such differences are critical in understanding the pathophysiology of CML across age groups and may have significant implications for diagnostic and therapeutic strategies (20). Moreover, research has revealed that in addition to the genetic aberrations differentiating pediatric and adult CML, there are also distinct variations in the signaling pathways involved. These differences in signaling mechanisms further underscore the complexity of CML pathogenesis across different age groups and highlight the need for age-specific approaches in both research and treatment of this disease (21). There are many unanswered aspects of TKI use in pediatric CML. The effectiveness of TKIs is directly related to their concentration in plasma, with better outcomes demonstrated in patients having a plasma concentration of at least 1000 ng/ml; however, this is only evidenced in the adult population and remains unexplored in pediatric CML patients (6). Additionally, prognostication criteria such as the National Comprehensive Cancer Network (NCCN) guidelines or the European Leukemia Net (ELN) are used in pediatric CML patients based on their validation for the adult CML population (6). As children have an immature skeleton and rapid growth phases, indefinite TKI therapy affects growth. Moreover, the duration of treatment with TKI in the pediatric CML population is considerably longer than in adults (5). This difference is even more clinically significant regarding drug safety, as the side effects of TKI are not well-studied beyond 15 years. The long-term side effect profile may differ from those of adults (5). TKI therapy discontinuation has been investigated in the adult CML population with favorable outcomes. However, it remains unexplored prospectively in children (22).

Rapidly accumulating data on the use of TKI in pediatric CML is emerging (23–25). Newer TKIs such as dasatinib and Nilotinib are increasingly being prescribed as upfront therapies in pediatric CML (26–28). Owing to the low prevalence of CML within the pediatric demographic, along with trials scrutinizing the impact of novel therapeutic approaches constrained to smaller cohorts, the characterization of efficacy and safety profiles of disparate TKI generations, as well as a thorough comparative analysis of the risk-benefit ratio between TKIs and SCT specifically in a pediatric context, remains nebulous. The objective of this narrative review was to discuss a balance between the effectiveness of TKIs with the adverse effect profile within the context of pediatric CML.

## Methods

We used Medline, OVID, and Web of Science to identify all prospective randomized clinical trials reporting the efficacy and safety of TKI in pediatric CML (< 18 years). We excluded observational studies, non-randomized clinical trials, studies describing patients aged > 18 years; those with contraindications to the use of TKI; the presence of another active malignancy, or the use of systemic therapy for another malignancy within three years (except local/regional therapy with curative intent). We used well-defined keywords in literature search (“pediatric chronic myeloid leukemia” OR “pediatric CML” OR “chronic myeloid leukemia in children” OR “adolescent chronic myeloid leukemia” OR “adolescent CML” OR “chronic myeloid leukemia in adolescents” AND “tyrosine kinase inhibitors” OR “TKI” OR “imatinib” OR “dasatinib” OR “nilotinib” OR “ponatinib” OR “bosutinib”. We aggregated the efficacy and safety data from the included RCTs, focusing on clinical and demographic variables and outcomes relevant to the safety and efficacy of TKI in the pediatric population with CML. The narrative review highlights key aspects of patient responses and potential adverse events, offering a comprehensive view of TKI treatment profiles.

## Discussion

### Patients characteristics

A total of 16 trials were included in this review, representing a collective of 887 pediatric CML patients. One study reported only safety outcomes and was removed from the efficacy analysis (n_(efficacy studies)_=15, n_(efficacy patients)_=718), while another reported only efficacy results and was removed from the safety analysis (n_(safety studies)_=15, n_(safety patients)_=699 patients). The median population age in the studies ranged from 6.5 to 14 years old (average 11.3 years old). Where data was available at baseline, the median white blood cell count was 234 x109/uL [range 19 to 378 x109/µL; reported in 9 of 16 studies]. The median hemoglobin level was 9.05 g/dL [range 5.6 to 10.8 g/dL; reported in 7 of 16 studies], and the median platelet count was 431.5 x109/µL, [range 31 to 594 x109/µL; reported in 6 of 16 studies]. Individual study data from the added RCTS are summarized in **Table 1**; **Supplementary Table 1**. The efficacy data is summarized in **Table 2**.

**Table 1:** Summary of patient demographics, Tyrosine Kinase Inhibitor (TKI) details, safety, and efficacy data from the included clinical trials with single cohorts.

Study	CML patients (n) and Baseline Data	TKI Median duration of TKI in months [range]	Switch/Stop details	Efficacy Data		Adverse Drug Reactions - ADRs (n)	Notes
(29)	n = 9 **Population**: Imatinib-refractory cases **Age (Median):** 11.7 **Hgb:** > 8 g/dL	Dasatinib as 2^nd^ line **Not reported (NR)**	NR	There were three complete cytogenetic responses, three partial cytogenetic responses, and two partials/ minimal cytogenetic responses observed in evaluable patients with CML		**ADR (n)** • Potassium derangement (1) • Diarrhea (2) • GI Bleeding (1) • Pyrexia (13) • AEs related to bilirubin increases (17) Edema (1)	• From total 39 patients, only 9 are CML • Time cut-off at cytogenetic response evaluation was not specified • Detailed efficacy results are NR
(24)	n=44 **Population**: previously untreated CML **Age (n):** • <5 years old (5) • 6 – 18 years old (39) **CML risk score (n):** • Low (7) • Intermediate (8) • High (29) **Splenomegaly (n)**: 37 **WBC (median; [range]):** 310; [16-762] **Hgb (median; [range]):** 8.8; [6.6-13.2]	Imatinib as 1^st^ line **27 [2 – 77]**	**n=13/44** • Unsatisfactory therapeutic effect (cytogenetic refractoriness): n =1 • Loss of complete hematologic response, CCyR, or molecular response: n= 3 • Non-achievement of major molecular response [MMR]: n = 5 • ADRs; n = 2 • AlloSCT, n= 2	**Parameter reported**		**ADR (n)** • Anemia (10) • Thrombocytopenia (14) • Neutropenia (39) • Hepatotoxicity (6) • Hypophosphatemia (8) • Nausea/Vomiting (19) • Diarrhea (9) • Periorbital Edema (7) • Headache (8) • Peripheral Neuropathy (6) • MSK pain (15) • Peripheral Edema (3) • Weight loss (2) • Fatigue (7) • Increased alkaline phosphatase (2)	OS: NR
				**CCYR at 12 months**	61%		
				**MMR at 12 months**	83%		
				**MMR at 18 months**	31%		
				**Estimated PFS at 36 months**	98%		
(30)	n=44 **Population**: newly diagnosed CML **Age range:** 4 – 17 years old	Imatinib as 1^st^ line **NR**	None stopped	NR		**ADR** • Hyperparathyroidism (n=8) • Low vitamin D (n=15) • Increased osteocalcin levels (58%) • Lowered serum procollagen type l N propeptide (25%) • Increased serum C-terminal cross-linking telopeptide of collagen (57%) • Increased urine pyridinoline levels (10%) • Increased urine deoxypyridinoline levels (9%)	Only safety parameters were reported
(31)	n=64 **Population**: previously untreated CML **Age (median; [range]):** 13 years old [1-18] **CML risk score (n):** • Low (34) • Intermediate (23) • High (7) **WBC (median; [range]):** 158; [5.9 - 539] **Hgb (median; [range]):** 9.3; [5.1 – 13.8] **PLT (median; [range]):** 380; [80 – 11,400] **Median blasts:** 3%	Imatinib as 1^st^ line **NR**	NR	• **CCYR at 12 months**: 78.3% • **PFS at 36 months:** 56.8% • **OS at 36 months:** 94.5%		**ADR (n) from cohort 1 only:** • Anemia (42) • Thrombocytopenia (11) • Neutropenia (18) • Hepatotoxicity (2) • Growth restriction (19) • Diarrhea (6) • Dyspepsia(5) • Constipation (3) • Cutaneous ADRs (44) • MSK pain (19) • Edema/weight gain (25) • Fatigue (8) • Asthenia (9) • Skin rash (7) • Oral ulcers (6)	MMR was NR
(32)	N=14 **Median age**: 9.5 Y **Median WBC:** 206.1 (48.7-775) **Median Hgb**: 10.2 (7.5-13.5) **Median PLT**: 460 (112-1455) - Newly- diagnosed (within the early chronic-phase) OR - Failed to respond to previous treatment (i.e., late chronic-phase)	Imatinib as 1^st^ and 2^nd^ line **22.5 [NR]**	**Reasons included:** - Disease progression (n=1) - Severe ADRs (n=1)	• **CCYR at 24 months**: 85.7% • **PFS at 30 months:** 85.71% • **OS (unclear if also at 30 months or not):** 86%		**ADR (n):** • Anemia (4) • Nausea/Vomiting (14) • Abdominal pain (3) • MSK pain (11) • Weight gain (8) • Dyspnea (2)	
(33)	N=11 **Median age**: 9.5 years old [5-17] Imatinib and/or dasatinib resistant CML in chronic or acute phases	Nilotinib as 2^nd^ line **24 [NR]**	**Reasons included:** - New cancer therapy (n=6) - Increasing blasts (n=1) -ADRs (n=1)	• **CCYR at 12 months**: 40% • **MMR at 18 months:** 27.3%		**ADR (n):** • Neutropenia (2) • Hepatotoxicity (8) • Nausea/Vomiting (7) • Diarrhea (2) • Abdominal pain (3) • Cutaneous ADRs (8) • Infections (4) • Headache (4) • MSK pain (3) • Pain in extremity (3) • Rhinitis (3)	PFS and OS are NR
(34)	N=14 **Median age**: 14 [3 – 20]	Imatinib as 2^nd^ line **12 [NR]**	None stopped	• **CCYR at 12 months**: 83% • **CCYR at 24 months:** 62% • **OS:** 92.86%		**ADR (n):** • Hepatotoxicity (2) • Nausea/Vomiting (3) • Diarrhea (1) • MSK pain (1)	MMR and PFS are NR
(23)	N=51 **Population**: previously untreated chronic phase CML **Age (median; [range]):** 11.8 years old [2.3 – 19.1] **WBC (median; [range]):** 95.4; [3.3–618] **PLT (median; [range]):** 53; [65 – 3,620] **Median blasts:** 1% [1 -8]	Imatinib as 1^st^ line **36 [NR]**	25 patients stopped for AlloSCT	• **CCYR at 12 months**: 38% • **PFS:** 72% • **OS:** 92%		**ADR (n):** • Anemia (7) • Thrombocytopenia (8) • Neutropenia (16) • Hepatotoxicity (3) • Hyperglycemia (1) • Potassium disturbance (3) • Hypophosphatemia (1) • Nausea/Vomiting (1) • Diarrhea (1) • Abdominal pain (3) • Headache (1) • MSK pain (6) • Edema (2) • Weight gain (2)	MMR was NR

CCYR, Complete Cytogenic Response; MMR, Major molecular remission; PFS, Progression-free survival; OS, Overall survival; Hgb, Hemoglobin (g/dL); WBC, White blood cell (x 10^9^/L); PLT, Platelets (x10^9^/L); ADR, Adverse drug reaction; CHF, Congestive heart failure; MSK, Musculoskeletal; CML, Chronic myeloid leukemia; NR, Not reported; TKI, Tyrosine Kinase Inhibitor; AlloSCT, allogeneic hematopoietic stem cell transplantation.

**Table 2:** Reporting of efficacy outcomes in included studies.

Reporting Categories	BCR::ABL reporting (N=15)	CCYR Reporting (N=15 studies)				MMR Reporting (N=15 studies)		
		At 12 months	At 24 months	At 36 months		At 12 months	At 18 months	At 24 months
**Not reported**	11	3	12	15		11	11	10
**Reported for all cohorts**	2	10	3	0		2	4	4
**Reported for one cohort only**	2	2	0	0		2	0	1
**Reporting Categories**	**Survival reporting (N=15)**							
	**PFS**				**OS**			
**Not reported**	5				4			
**Reported for all cohorts**	9				9			
**Reported for one cohort only**	1				2			

CCYR, Complete Cytogenic Response; MMR, Major molecular remission; PFS, Progression-free survival; OS, Overall survival.

### Choosing initial treatment and efficacy

Tyrosine kinase inhibitors (TKIs) have revolutionized the management of CML pediatrics. Allogeneic hematopoietic stem cell transplantation (Allo-HSCT) was considered the standard of care in pediatrics, with overall survival ranging between 60 and 80% (35). However, the approval of imatinib and second- generation TKIs dasatinib and nilotinib in pediatric CML expanded the therapeutic options for treatment in this population.

Among the included trials, TKIs were used as first-line treatment in 65% of the studies, with Imatinib being the most commonly used drug (as monotherapy in 50% of studies). Dasatinib and nilotinib were used in 17% and 11% of studies, respectively, while a small percentage of patients (6%) received a combination of Imatinib and chemotherapy. Reporting of efficacy outcomes in the studies was a major concern that challenged the accumulation of results, as shown in **Table 1**; **Supplementary Table 1**. In the studies that reported a BCR::ABL response of <10% (n= 4 studies), the percentage of patients ranged between 60% and 78%, while for complete cytogenetic response (CCyR) at 24 months (n = 3 studies), the percentage of patients ranged from 62% to 94%. Progression free survival (PFS; n= 10 studies) ranged from 56.8% to 100%; however, the timepoint for PFS analysis was not standardized among studies (ranging from 36 to 48 months).

Despite the approval of dasatinib and nilotinib, Imatinib remains the most studied TKI in pediatrics, leading to it being the preferred medication for first line treatment of CML in pediatric patients (35). Furthermore, a retrospective cohort study in pediatric patients (median age of 10.5 years) compared the outcomes in patients treated with HSCT prior to the introduction of Imatinib and TKIs Vs. after 2002. Post-HSCT, OS was 64% at 6 years Vs. 90% at 3 years with TKIs (statistically significant with p-value of 0.008) (36). Moreover, the results reported in the eligible trials included were comparable to results from adult studied. The 7-year-follow-up IRIS study reported 83% CCyR, 93% PFS and 88% OS among adult CML patients receiving Imatinib (37).

### Safety of TKIs in pediatric CML

Lifelong TKIs administration and the associated toxicities in pediatrics remain a concern. The available data for safety included adverse events (AEs) experienced by patients, which were reported for 851 patients undergoing treatment (n_(safety studies)_ = 15). The most common AEs were anemia (n_(patients)_ = 225), thrombocytopenia (n_(patients)_ = 159), and neutropenia (n_(patients)_ = 254). Other common adverse events include hepatotoxicity (n_(patients)_ = 77) and cutaneous side effects (n_(patients)_ = 149).

The safety profiles of dasatinib and nilotinib were consistent with those reported in adults, except that no cases of peripheral arterial occlusive disease, ischemic heart or ischemic cerebrovascular disease, cardiac arrhythmias, pleural effusion, pulmonary arterial hypertension, pneumonitis, gonadal dysfunction, pancreatitis, loss of visual acuity, or conjunctival hemorrhage were reported. With dasatinib, the concerns for pleuro-pulmonary toxicities seen in adults are quite rare in pediatrics, with most of the few cases reported being grade 2 or lower. Some cardiovascular complications were observed with second-generation TKIs, such as QTc prolongation with nilotinib (n_(patients)_ = 8) and congestive heart failure with dasatinib (n_(patients)_ = 4). Gastrointestinal side effects were reported in all TKIs but occurred more frequently in imatinib patients. Nausea and vomiting were reported in 126 patients receiving Imatinib, versus 54 and 27 patients receiving dasatinib and nilotinib, respectively. Diarrhea occurred in 40 patients on Imatinib compared with 20 and 2 patients in the dasatinib and nilotinib cohorts, respectively.

Other AEs included, impaired bone and growth development reported with Imatinib (n_(patients)_ = 70) and dasatinib (n_(patients)_ = 5). Similar incidences of headache were reported with Imatinib (n_(patients)_ = 36) and nilotinib (n_(patients)_ = 31) compared with dasatinib (n_(patients)_ = 13). A higher incidence of musculoskeletal pain was observed in imatinib patients (n_(patients)_ = 155), followed by dasatinib (n_(patients)_ = 19), and least with nilotinib (n_(patients)_ = 3). Treatment AEs leading to drug discontinuation were reported in 22 patients from 6 studies, however, the exact AE leading to drug discontinuation was not reported in the studies. As the maximum follow-up period in pediatric studies thus far remains relatively short; further investigation into the chronic effects of TKIs in children is imperative before drawing definitive conclusions.

One of the challenges that remain is the assessment of long-term safety of TKI use in children with CML. As evident from data on adult CML and TKI use, some toxicities might appear early on in the course of treatment and improve over time, whereas many toxicities may appear after years and be of more clinical significance. This include but are not limited to pleural effusions and vascular events (38). Growth restriction of deceleration represent a critical area of concern for potential long-term adverse effects in pediatric CML, a phenomenon less likely to manifest in adult populations. Future longitudinal research explicitly examining the long-term safety of Tyrosine Kinase Inhibitors (TKIs) in pediatric CML populations would be invaluable. Such research is increasingly imperative, given the escalating utilization of TKIs in treating pediatric CML.

### Discontinuation of TKIs in pediatrics

Discontinuation of TKI therapy in adults has been well studied, but TKI discontinuation in pediatric CML still relies on experience in adults (22). Understanding the mechanism, efficacy rates, pharmacokinetics and pharmacodynamics, short-term and long-term complications, and treatment-free remissions with TKI therapy in the pediatric CML population is essential. From a preliminary data of 102 pediatric CML patients, JT Tauer et al. identified growth retardation as a significant adverse effect associated with TKI therapy. These patients received Imatinib as an upfront therapy for a median duration of 9 months (39). The impact of TKIs on bone growth and cellular functional capacity is anticipated to be more pronounced in pediatric patients, attributed to their ongoing growth and development. This underscores the urgent need for more extensive data to thoroughly assess the long-term safety of TKIs. Such data is vital for a comprehensive evaluation of the risk-benefit profile of TKIs, especially considering these age-specific adverse effect which may guide the optimal duration of therapy (39).

Data from the International Registry of Childhood Chronic Myeloid Leukemia provides information on imatinib discontinuation in a cohort of patients under 18 years old (40). The findings of 18 patients who had sustained deep molecular response (DMR) followed by imatinib discontinuation were reported. After discontinuation, the molecular-free remission rate was 61%, 56%, and 56% at 6, 12, and 36 months, respectively. The results of 22 pediatric patients with CML were reported by Shima et al. The treatment-free remission (TFR) rate at 12 months was 50.0% (90% confidence interval: 31.7%–65.8%). However, 11 patients experienced loss of MMR within four months after TKI discontinuation and resumed TKI as originally prescribed (41). Moreover, the STOP IMAPED study reported the results of four patients who maintained DMR at 24 and 36 months after stopping TKI, whereas ten patients relapsed (42).

In a recent retrospective cohort study by Satishkumar et al., it was shown that among 11 pediatric patients who were treated with Imatinib for five years and sustained molecular response for at least two years, 8 (72%) successfully retained TFR (43). While the other three patients had disease recurrence, they achieved MMR upon restarting Imatinib. Given the chronic nature of CML and the potential impact that ongoing treatment may have on pediatrics’ quality of life, this is encouraging. The mentioned study, however, might be the only one offering insight into TFR in pediatric CML patients.

### Challenges and literature gaps

There were several challenges that hindered accumulating pediatric data within a study. One of the major challenges was heterogeneity, both in population characteristics and outcome definitions. Furthermore, many studies explored the pharmacokinetics of TKIs, which, although essential, did not provide accurate results on their efficacy and long-term safety in pediatrics. A consensus on clinically significant outcome reporting in pediatric CML research, including TFR, may be useful in reducing these challenges for future systematic reviews and meta-analysis.

## Conclusions

There is limited experience with CML pediatrics due to the low incidence of CML in this population. Therefore, to establish standardized guidelines for the therapeutic management of this population, information from prospective clinical trials and real-life clinical practice is needed. Compared to the other TKIs, there is more experience with imatinib efficacy and toxicity profile in pediatric patients. The safety profile of TKIs was consistent with the known safety profile in adults. With the availability of three TKIs as first line options, other factors should be considered when selecting a first- line TKI, including drug formulation, administration, comorbidities, and financial issues. Careful monitoring of adverse events, especially in growing children, should be considered with long- term follow-up clinical trials.

## Author contributions

FA: Conceptualization, Data curation, Formal analysis, Investigation, Methodology, Project administration, Writing – original draft, Writing – review & editing. MB: Data curation, Formal analysis, Writing – original draft. RG: Data curation, Formal analysis, Writing – original draft. LF: Data curation, Validation, Writing – review & editing. NO: Data curation, Writing – original draft. AN: Data curation, Writing – original draft. MA: Data curation, Writing – original draft. KM: Data curation, Writing – original draft. NK: Data curation, Writing – original draft. MY: Conceptualization, Methodology, Project administration, Supervision, Validation, Writing – original draft, Writing – review & editing.
