# Supplementary material for: Tyrosine Kinase Inhibitors in pediatric chronic myeloid leukemia: a focused review of clinical trials
Source: Front Oncol. 2023 Dec 20;13:1285346. doi: 10.3389/fonc.2023.1285346 (PMC10769570; doi:10.3389/fonc.2023.1285346)
Supplement: Supplementary file 1 [file Table_1.docx]

| **Summary of patient demographics, Tyrosine Kinase Inhibitor (TKI) details, safety, and efficacy data from the included clinical trials with multiple cohorts** | | | | | | | | | | | | | | | | | | | | | | | | | |
| --- | --- | --- | --- | --- | --- | --- | --- | --- | --- | --- | --- | --- | --- | --- | --- | --- | --- | --- | --- | --- | --- | --- | --- | --- | --- |
| **Study** | **CML patients (n) and Baseline Data** | | | | | | | | **TKI**  **Median duration of TKI in months [range]** | **Switch/ Stop details** | **Efficacy Data** | | | | | | | | **Adverse Reactions - ADRs (n)** | | | | | | **Notes** |
| **(Hijiya et al., 2019)** | **Cohort 1** | | | | **Cohort 2** | | | | **Cohort 1:**  Nilotinib as 2^nd^ line  **22.1 [0.5 – 44]**  **Cohort 2:**  Nilotinib as 1^st^ line  **22.3 [0.7 – 38.7]** | NR | **Cohort** | | | **1** | | | | **2** | **Cohort** | | | **1** | | **2** |  |
|  | Age 1 to 12 years old: 18 patients  Age 13 - 18 years old: 40 patients | | | | | | | |  |  | **CCYR at 12 months** | | | 81.82% | | | | 84% | **Thrombocytopenia** | | | 1 | | 8 |  |
|  |  |  |  |  |  |  |  |  |  |  |  |  |  |  |  |  |  |  | **QTc prolongation** | | | 5 | | 3 |  |
|  | n = 33 | | | | n = 25 | | | |  |  | **CCYR at 24 months** | | | 81.82% | | | | 84% | **Hepatotoxicity** | | | 20 | | 16 |  |
|  | R/I to either Imatinib or dasatinib | | | | Newly diagnosed | | | |  |  |  |  |  |  |  |  |  |  | **Hyperglycemia** | | | 2 | | 0 |  |
|  |  |  |  |  |  |  |  |  |  |  | **MMR at 18 months** | | | 57.58% | | | | 64% | **Hypercholesterolemia** | | | 3 | | 2 |  |
|  |  |  |  |  |  |  |  |  |  |  |  |  |  |  |  |  |  |  | **Nausea / vomiting** | | | 13 | | 14 |  |
|  |  |  |  |  |  |  |  |  |  |  | **MMR at 24 months** | | | 57.58% | | | | 68% | **Cutaneous ADRs** | | | 15 | | 15 |  |
|  |  |  |  |  |  |  |  |  |  |  |  |  |  |  |  |  |  |  | **Infections** | | | 16 | | 13 |  |
|  |  |  |  |  |  |  |  |  |  |  | **Estimated PFS at 24 months** | | | 96.3% | | | | 91.2% | **Headache** | | | 13 | | 14 |  |
|  |  |  |  |  |  |  |  |  |  |  |  |  |  |  |  |  |  |  | **Pyrexia** | | | 13 | | 1 |  |
|  |  |  |  |  |  |  |  |  |  |  | **Estimated OS at 24 months** | | | 96.9% | | | | | **Fatigue** | | | 0 | | 6 |  |
|  |  |  |  |  |  |  |  |  |  |  |  |  |  |  |  |  |  |  | **Edema** | | | 1 | | 3 |  |
|  |  |  |  |  |  |  |  |  |  |  |  |  |  |  |  |  |  |  | **AEs related to bilirubin increases** | | | 17 | | 4 |  |
| **(Millot et al., 2006)** | **Cohort 1** | | | | **Cohort 2** | | | | Imatinib as 1^st^ line  **28 [2 – 48]** | **Cohort 1 (n=12/22)** | **Cohort** | | | **1** | | | | **2** | **ADR (n):**   - Anemia (1) - Thrombocytopenia (6) - Neutropenia (10) - Hepatotoxicity (2) - Nausea/Vomiting (7) - Diarrhea (1) - Cutaneous ADRs (4) - Infections (5) - Headache (1) - Edema (1) | | | | | | -MMR and PFS are NR  -ADRs were reported for cohort 1 and 2 as one |
|  | n = 22 | | | | n = 8 | | | |  | - Shifting to AlloSCT for progression to accelerated phase (n=1) - Achieved CHR or PHR (n=3) - Undetectable transcript in (n=6) | **CCYR at 12 months** | | | 60% | | | | 29% |  |  |  |  |  |  |  |
|  | **Age (Median; [range]):** 13 years old [1 – 17.5] | | | | | | | |  |  | **OS** | | | 95% | | | | 75% |  |  |  |  |  |  |  |
|  | Newly diagnosed CML in chronic phase | | | | Newly diagnosed CML in advanced phase | | | |  |  |  |  |  |  |  |  |  |  |  |  |  |  |  |  |  |
|  |  |  |  |  |  |  |  |  |  | **Cohort 2 (n=6/8)** |  |  |  |  |  |  |  |  |  |  |  |  |  |  |  |
|  |  |  |  |  |  |  |  |  |  | - AlloSCT in patients with CHR or PHR (n=4) - Blast crisis after HSCT (n=1) - Occurrence of cerebral aspergillosis after AlloSCT (n=1) |  |  |  |  |  |  |  |  |  |  |  |  |  |  |  |
| **Study** | **CML patients (n) and Baseline Data** | | | | | | | | **TKI**  **Median duration of TKI in months [range]** | **Switch/ Stop details** | **Efficacy Data** | | | | | | | | **Adverse Reactions - ADRs (n)** | | | | | | **Notes** |
| **(Gore et al., 2018)** | **Cohort 1** | | | | | **Cohort 2** | | | **Cohort 1:**  Dasatinib as 2^nd^ line  **49.9 [1.9 - 90.2]**  **Cohort 2:**  Dasatinib as 1^st^ line  **42.23** **[0.1 - 75.5]** | **Cohort 1 (n=15/29)** | **Cohort** | | | | | **1** | | **2** | **Cohort** | | | **1** | | **2** | From total 145 patients, 113 were included |
|  | **Age (Median):** 13.7 | | | | | **Age (Median):** 12.3 | | |  | - Progressive disease (n=5) - Patient withdrawal (n=3) - Maximum clinical benefit (n=2) - Patient non-compliance (n=1) - Dasatinib hypersensitivity (n=0) - Others (n=4) | **CCYR at 12 months** | | | | | 92% | | 94% | **Anemia** | | | 1 | | 2 |  |
|  |  |  |  |  |  |  |  |  |  |  | **CCYR at 24 months** | | | | | 94% | | NR | **Neutropenia** | | | 2 | | 0 |  |
|  | n = 29 | | | | | n = 84 | | |  |  | **MMR at 12 months** | | | | | 83% | | NR | **CHF** | | | 1 | | 3 |  |
|  |  |  |  |  |  |  |  |  |  |  | **MMR at 18 months** | | | | | 31% | | NR | **Hepatotoxicity** | | | 1 | | 0 |  |
|  | R/I to Imatinib | | | | | Newly diagnosed | | |  |  | **Estimated PFS at 36 months** | | | | | 98% | | NR | **Growth restriction** | | | 3 | | 2 |  |
|  |  |  |  |  |  |  |  |  |  |  |  |  |  |  |  |  |  |  | **Nausea / vomiting** | | | 9 | | 17 |  |
|  |  |  |  |  |  |  |  |  |  |  | **Estimated OS at 48 months** | | | | | 96% | | 100% | **Diarrhea** | | | 4 | | 15 |  |
|  |  |  |  |  |  |  |  |  |  |  |  |  |  |  |  |  |  |  | **Cutaneous ADRs** | | | 4 | | 16 |  |
|  |  |  |  |  |  |  |  |  |  |  |  |  |  |  |  |  |  |  | **Infections** | | | 1 | | 0 |  |
|  |  |  |  |  |  |  |  |  |  |  |  |  |  |  |  |  |  |  | **Musculoskeletal pain** | | | 5 | | 8 |  |
|  |  |  |  |  |  |  |  |  |  | **Cohort 2 (n=23/84)** |  |  |  |  |  |  |  |  | **Shortness of breath** | | | 3 | | 0 |  |
|  |  |  |  |  |  |  |  |  |  | - Progressive disease (n=6) - Patient withdrawal (n=3) - Maximum clinical benefit (n=1) - Patient non-compliance (n=0) - Dasatinib hypersensitivity (n=1) - Others (n=12) |  |  |  |  |  |  |  |  | **Hematochezia** | | | 0 | | 1 |  |
|  |  |  |  |  |  |  |  |  |  |  |  |  |  |  |  |  |  |  | **Edema** | | | 1 | | 2 |  |
|  |  |  |  |  |  |  |  |  |  |  |  |  |  |  |  |  |  |  | **Hemorrhage** | | | 3 | | 8 |  |
|  |  |  |  |  |  |  |  |  |  |  |  |  |  |  |  |  |  |  | **Chest pain** | | | 0 | | 1 |  |
|  |  |  |  |  |  |  |  |  |  |  |  |  |  |  |  |  |  |  | **Periorbital cellulitis** | | | 0 | | 1 |  |
|  |  |  |  |  |  |  |  |  |  |  |  |  |  |  |  |  |  |  | **Recurrent leukemia** | | | 0 | | 1 |  |
|  |  |  |  |  |  |  |  |  |  |  |  |  |  |  |  |  |  |  | **Febrile neutropenia** | | | 0 | | 1 |  |
|  |  |  |  |  |  |  |  |  |  |  |  |  |  |  |  |  |  |  | **Hypersensitivity** | | | 0 | | 1 |  |
| **(Millot et al., 2019)** | **Cohort 1** | | | **Cohort 2** | | | | | 1^st^ line in all patients  **Cohort 1:**   - Imatinib (n=18) - **NR** - 2^nd^ G TKI (n=1)   **NR**  **Cohort 2:**   - Imatinib (n=7)   **NR**   - CT + imatinib or 2^nd^ G TKI (n=10) - **NR** | **For cohort 2, not reported.**  **In cohort 1**, 9 patients switched/discontinued TKI, reasons included:   - Loss of CHR (n=1) - Progression to lymphoid blast phase (n=2) - Myeloid blast phase (n=1) - Loss of molecular response (n=2) - No achievement of complete cytogenetic response [CCyR] (n=1) - Treating physicians' choice (n=2) | **Cohort** | | **1** | | | | **2** | | NR | | | | | | -CCYR and PFS were NR  - Safety data was NR |
|  | n = 19 | | | n = 17 | | | | |  |  |  |  |  |  |  |  |  |  |  |  |  |  |  |  |  |
|  | CML-acute phase | | | CML-blast phase | | | | |  |  | **MMR at 24 months** | | 84.21% | | | | 76.47% | |  |  |  |  |  |  |  |
|  | **Age (Median; [range]):** 12.7 years old [3 – 18] | | | **Age (Median; [range]):** 11.1 years old [5 – 17] | | | | |  |  |  |  |  |  |  |  |  |  |  |  |  |  |  |  |  |
|  | **WBC (median; [range]):**  269 [5-657] | | | **WBC (median; [range]):**  295 [37-653] | | | | |  |  | **OS at 5 years** | | 94% | | | | 74% | |  |  |  |  |  |  |  |
|  | **Hgb (median; [range]):**  8.7 [5.1-11.5] | | | **Hgb (median; [range]):**  7.4 [2.2-12.3] | | | | |  |  |  |  |  |  |  |  |  |  |  |  |  |  |  |  |  |
|  | **Splenomegaly:**  n=18 | | | **Splenomegaly:**  n=13 | | | | |  |  |  |  |  |  |  |  |  |  |  |  |  |  |  |  |  |
|  | **Median blasts in blood:** 6% | | | **Median blasts in blood:** 32% | | | | |  |  |  |  |  |  |  |  |  |  |  |  |  |  |  |  |  |
| **Study** | **CML patients (n) and Baseline Data** | | | | | | | | **TKI**  **Median duration of TKI in months [range]** | **Switch/ Stop details** | **Efficacy Data** | | | | | | | | **Adverse Reactions - ADRs (n)** | | | | | | **Notes** |
| **(Suttorp et al., 2018)** | **Cohort** | | | | | | | | Imatinib  **Cohorts 1 and 2:**  **15 [0 – 102]**  **Cohort 3:**  **36 [NR]** | **Cohort 1:**   - Imatinib failure (n=38)   - Due to lack of response (n=29)   - Due to intolerance (n=9) - AlloSCT (n=12) - Stopping after prolonged response, (n=7) | **Parameter** | | | | | | **Results** | | **ADR (n) from cohort 1 only:**   - Anemia (98) - Thrombocytopenia (40) - Neutropenia (72) - Hepatotoxicity (11) - Growth restriction (51) - Nausea/Vomiting (63) - Diarrhea (14) - Periorbital Edema (4) - Cutaneous ADTs (26) - Infections (38) - Headache (18) - MSK pain (62) - Edema (7) - Fatigue (10) - Alopecia (1) | | | | | | -Efficacy was NR for cohorts 2 and 3.  -Safety was reported in cohort 1 only |
|  | **1** | | **2** | | | | | **3** |  |  |  |  |  |  |  |  |  |  |  |  |  |  |  |  |  |
|  | **Number of patients** | | | | | | | |  |  |  |  |  |  |  |  |  |  |  |  |  |  |  |  |  |
|  | 140 | | 3 | | | | | 5 |  |  | **CCYR at 12 months** | | | | | | 63% | |  |  |  |  |  |  |  |
|  | **Population** | | | | | | | |  |  | **MMR at 12 months** | | | | | | 86% | |  |  |  |  |  |  |  |
|  | CML chronic phase | | CML acute phase | | | | | CML blast phase |  |  | **PFS at 18 months** | | | | | | 97% | |  |  |  |  |  |  |  |
|  | **Dosing regimen** | | | | | | | |  |  | **OS at 18 months** | | | | | | 100% | |  |  |  |  |  |  |  |
|  | Imatinib 260–300 mg/m^2^ | | Imatinib 400 mg/m^2^ | | | | | CT then Imatinib  500 mg/m2 |  |  |  |  |  |  |  |  |  |  |  |  |  |  |  |  |  |
|  |  |  |  |  |  |  |  |  |  | **Cohort 2:**  2 patients were switched, but reasons are NR |  |  |  |  |  |  |  |  |  |  |  |  |  |  |  |
|  | **Age (median years old [range])** | | | | | | | |  |  |  |  |  |  |  |  |  |  |  |  |  |  |  |  |  |
|  | 13.3  [1.3-18] | | 11.1  [8.4-17.4] | | | | | 6.5  [5-13.9] |  |  |  |  |  |  |  |  |  |  |  |  |  |  |  |  |  |
|  | **Splenomegaly (n)** | | | | | | | |  | **Cohort 3:**  2 patients, both died after AlloSCT |  |  |  |  |  |  |  |  |  |  |  |  |  |  |  |
|  | 97 | | 3 | | | | | 2 |  |  |  |  |  |  |  |  |  |  |  |  |  |  |  |  |  |
|  | **WBC (median)** | | | | | | | |  |  |  |  |  |  |  |  |  |  |  |  |  |  |  |  |  |
|  | 205 | | 264 | | | | | 19 |  |  |  |  |  |  |  |  |  |  |  |  |  |  |  |  |  |
|  | **Hgb (median)** | | | | | | | |  |  |  |  |  |  |  |  |  |  |  |  |  |  |  |  |  |
|  | 9.6 | | 6.1 | | | | | 5.6 |  |  |  |  |  |  |  |  |  |  |  |  |  |  |  |  |  |
|  | **PLT (median)** | | | | | | | |  |  |  |  |  |  |  |  |  |  |  |  |  |  |  |  |  |
|  | 483 | | 594 | | | | | 31 |  |  |  |  |  |  |  |  |  |  |  |  |  |  |  |  |  |
| **(Muramatsu et al., 2011)** | **Cohort 1** | | | | **Cohort 2** | | | | **TKI in Cohort 1 only:**  Imatinib as 1^st^ line  **36 [NR]**  **Cohort 2 was AlloSCT without TKI** | None | Cohort 1 only:   - **CCYR at 12 months**: 54.6% - **MMR at 12 months:** 90.9% - **MMR at 36 months:** 36.4% | | | | | | | | **ADR (n) from cohort 1 only:**   - Anemia (1) - Thrombocytopenia (3) - Neutropenia (1) - Cutaneous ADRs (1) - MSK pain (2) | | | | | | Efficacy for cohort 2 is not clearly stated |
|  | n=12 | | | | n=16 | | | |  |  |  |  |  |  |  |  |  |  |  |  |  |  |  |  |  |
|  | **Age (Median years old; [range])** | | | | | | | |  |  |  |  |  |  |  |  |  |  |  |  |  |  |  |  |  |
|  | 9 [2 – 14] | | | | 9 [3 – 14] | | | |  |  |  |  |  |  |  |  |  |  |  |  |  |  |  |  |  |
|  | **Splenomegaly (median cm below costal margin; [range])** | | | | | | | |  |  |  |  |  |  |  |  |  |  |  |  |  |  |  |  |  |
|  | 4 [0 – 16] | | | | 6 [0 – 22] | | | |  |  |  |  |  |  |  |  |  |  |  |  |  |  |  |  |  |
|  | **WBC (median; [range])** | | | | | | | |  |  |  |  |  |  |  |  |  |  |  |  |  |  |  |  |  |
|  | 111 [41 – 484] | | | | 234 [21 – 850] | | | |  |  | **Cohort** | | | | **1** | | | **2** |  |  |  |  |  |  |  |
|  | **Hgb (median; [range])** | | | | | | | |  |  | **PFS at 5 years** | | | | 100% | | | 87.5% |  |  |  |  |  |  |  |
|  | 10.8 [5.9 – 13.1] | | | | 10.3 [5.9 – 12.2] | | | |  |  |  |  |  |  |  |  |  |  |  |  |  |  |  |  |  |
|  | **PLT (median; [range])** | | | | | | | |  |  |  |  |  |  |  |  |  |  |  |  |  |  |  |  |  |
|  | 558 [150 – 944] | | | | 403 [115 – 1380] | | | |  |  |  |  |  |  |  |  |  |  |  |  |  |  |  |  |  |
|  |  | | | |  | | | |  |  |  |  |  |  |  |  |  |  |  |  |  |  |  |  |  |
| **Study** | **CML patients (n) and Baseline Data** | | | | | | | | **TKI**  **Median duration of TKI in months [range]** | **Switch/ Stop details** | **Efficacy Data** | | | | | | | | **Adverse Reactions - ADRs (n)** | | | | | | **Notes** |
| **(Zwaan et al., 2013)** | **Cohort** | | | | | | | | Dasatinib as 2^nd^ line for all cohorts  **Cohort 1:**  **24.1 [NR]**  **Cohort 2:**  **3 [NR]**  **Cohort 3:**  **1.1 [NR]** | **Cohort 1: (n=6)**  AlloSCT | **Cohort** | **1** | **2** | | | | **3** | | **Cohort** | **1** | **2** | | **3** | |  |
|  | 1 | 2 | | | | | 3 | |  |  |  |  |  |  |  |  |  |  | **Anemia** | 12 | 15 | | 22 | |  |
|  | **Number of patients** | | | | | | | |  |  |  |  |  |  |  |  |  |  |  |  |  |  |  |  |  |
|  | 17 | 17 | | | | | 24 | |  |  | **CCYR at 12 months** | 82% | 100% | | | | 0% | | **Thrombo-cytopenia** | 11 | 16 | | 23 | |  |
|  | **Population** | | | | | | | |  |  |  |  |  |  |  |  |  |  |  |  |  |  |  |  |  |
|  | R/I to imatinib chronic phase | R/I to imatinib acute phase | | | | | R/R Ph-positive ALL or AML after $\geq$2 lines | |  |  |  |  |  |  |  |  |  |  | **Neutropenia** | 14 | 15 | | 22 | |  |
|  |  |  |  |  |  |  |  |  |  |  |  |  |  |  |  |  |  |  | **Nausea/ vomiting** | 7 | 8 | | 13 | |  |
|  | **Dosing regimen** | | | | | | | |  | **Cohort 2: (n=10)**  Resistant/ refractory disease | **MMR at 24 moths** | 47% | 25% | | | | 0% | | **Diarrhea** | 4 | 4 | | 2 | |  |
|  | Initial cohorts were started on 60 mg/m2 once daily, and subsequent cohorts received 80 mg/m2 once daily | | | | | | dasatinib 100 and 120 mg/m2 once daily | |  |  |  |  |  |  |  |  |  |  | **Cutaneous AE** | 8 | 4 | | 2 | |  |
|  |  |  |  |  |  |  |  |  |  |  |  |  |  |  |  |  |  |  | **Infections** | 2 | 0 | | 0 | |  |
|  |  |  |  |  |  |  |  |  |  |  |  |  |  |  |  |  |  |  | **Headache** | 6 | 3 | | 4 | |  |
|  |  |  |  |  |  |  |  |  |  | **Cohort 3: (n=19)**  -Drug-related toxicity (n=2)  - Resistant/ refractory disease (n=17) | **PFS** | 61% | Median 4.9 months | | | | Median 1.4 months | | **MSK pain** | 2 | 1 | | 3 | |  |
|  |  |  |  |  |  |  |  |  |  |  |  |  |  |  |  |  |  |  | **Back pain** | 2 | 0 | | 1 | |  |
|  |  |  |  |  |  |  |  |  |  |  |  |  |  |  |  |  |  |  | **Pain in extremity** | 4 | 1 | | 1 | |  |
|  |  |  |  |  |  |  |  |  |  |  | **OS** | 88% | Median 8.6 months | | | | Median 1.4 months | | **Pyrexia** | 2 | 1 | | 2 | |  |
|  | **Age (median years old [range])** | | | | | | | |  |  |  |  |  |  |  |  |  |  | **Fatigue** | 2 | 2 | | 1 | |  |
|  | 10 [NR] | | | | | | | |  |  |  |  |  |  |  |  |  |  | **Peripheral edema** | 2 | 0 | | 0 | |  |
|  |  |  |  |  |  |  |  |  |  |  |  |  |  |  |  |  |  |  | **Epistaxis** | 1 | 2 | | 0 | |  |
|  |  |  |  |  |  |  |  |  |  |  |  |  |  |  |  |  |  |  | **Malaise** | 2 | 0 | | 0 | |  |
|  |  |  |  |  |  |  |  |  |  |  |  |  |  |  |  |  |  |  | **Mouth ulcers** | 2 | 0 | | 0 | |  |
| **Study** | **CML patients (n) and Baseline Data** | | | | | | | | **TKI**  **Median duration of TKI in months [range]** | **Switch/ Stop details** | **Efficacy Data** | | | | | | | | **Adverse Reactions - ADRs (n)** | | | | | | **Notes** |
| **(Millot et al., 2014)** | **Cohort 1** | | | | **Cohort 2** | | | | Imatinib as 1^st^ line for all cohorts  **48 [NR]** | 1 patient switched to dasatinib due to toxicity | **Cohort** | | | | | **1** | | **2** | **ADR (n) – reported for all cohorts together:**   - Anemia (10) - Thrombocytopenia (14) - Neutropenia (39) - Hepatotoxicity (6) - Hypophosphatemia (8) - Nausea/Vomiting (19) - Diarrhea (9) - Periorbital Edema (7) - Headache (8) - Peripheral Neuropathy (6) - MSK pain (39) - Peripheral Edema (3) - Weight loss (2) - Fatigue (7) - Increased alkaline phosphatase (2) | | | | | |  |
|  | n=25 | | | | n=15 | | | |  |  | **CCYR at 12 months** | | | | | 76% | | 47% |  |  |  |  |  |  |  |
|  | BCR::ABL $\leq$ 10%  CML chronic phase (newly diagnosed) | | | | BCR::ABL>10%  CML chronic phase (newly diagnosed) | | | |  |  |  |  |  |  |  |  |  |  |  |  |  |  |  |  |  |
|  |  |  |  |  |  |  |  |  |  |  | **MMR at 12 months** | | | | | 48% | | 7% |  |  |  |  |  |  |  |
|  |  |  |  |  |  |  |  |  |  |  | **PFS** | | | | | 100% | | 61% |  |  |  |  |  |  |  |
|  | imatinib 260 mg/m2 daily | | | | | | | |  |  |  |  |  |  |  |  |  |  |  |  |  |  |  |  |  |
|  | **Median age [range]** | | | | | | | |  |  |  |  |  |  |  |  |  |  |  |  |  |  |  |  |  |
|  | 11.3 [0.8-16.7] | | | | 11.7 [1.9-17.3] | | | |  |  |  |  |  |  |  |  |  |  |  |  |  |  |  |  |  |
|  | **CML risk score** | | | | | | | |  |  |  |  |  |  |  |  |  |  |  |  |  |  |  |  |  |
|  | - Low: 5 - Intermediate: 7 - High: 13 | | | | - Low: 2 - Intermediate: 1 - High: 12 | | | |  |  |  |  |  |  |  |  |  |  |  |  |  |  |  |  |  |
|  | **Splenomegaly** | | | | | | | |  |  |  |  |  |  |  |  |  |  |  |  |  |  |  |  |  |
|  | n=5 [0-21] | | | | n=13 [0-21] | | | |  |  |  |  |  |  |  |  |  |  |  |  |  |  |  |  |  |
|  | **Median WBC count** | | | | | | | |  |  |  |  |  |  |  |  |  |  |  |  |  |  |  |  |  |
|  | 252 [16 – 482] | | | | 378 [44 – 762] | | | |  |  |  |  |  |  |  |  |  |  |  |  |  |  |  |  |  |
| ALL: Acute lymphoblastic leukemia; AML: Acute myeloid leukemia; CCYR: Complete Cytogenic Response; CT: Chemotherapy; CHR: Complete hematological remission; PHR: Partial hematological remission; MMR: Major molecular remission; PFS: Progression-free survival; OS: Overall survival; Hgb: Hemoglobin (g/dL); WBC: White blood cell (x 10^9^/L); PLT: Platelets (x 10^9^/L); ADR: Adverse drug reaction; CHF: Congestive heart failure; MSK: Musculoskeletal; CML: Chronic myeloid leukemia; NR: Not reported; R/I: Relapsed or intolerant; R/R: Relapsed or refractory; TKI: Tyrosine Kinase Inhibitor; AlloSCT: allogeneic hematopoietic stem cell transplantation | | | | | | | | | | | | | | | | | | | | | | | | | |

**REFERENCES**

Gore, L., Kearns, P.R., De Martino, M.L., Lee, De Souza, C.A., Bertrand, Y., Hijiya, N., Stork, L.C., Chung, N.G., Cardos, R.C., Saikia, T., Fagioli, F., Seo, J.J., Landman-Parker, J., Lancaster, D., Place, A.E., Rabin, K.R., Sacchi, M., Swanink, R., and Zwaan, C.M. (2018). Dasatinib in Pediatric Patients With Chronic Myeloid Leukemia in Chronic Phase: Results From a Phase II Trial. *J Clin Oncol* 36**,** 1330-1338.

Hijiya, N., Maschan, A., Rizzari, C., Shimada, H., Dufour, C., Goto, H., Kang, H.J., Guinipero, T., Karakas, Z., Bautista, F., Ducassou, S., Yoo, K.H., Zwaan, C.M., Millot, F., Aimone, P., Allepuz, A., Quenet, S., Hourcade-Potelleret, F., Hertle, S., and Sosothikul, D. (2019). Phase 2 study of nilotinib in pediatric patients with Philadelphia chromosome–positive chronic myeloid leukemia. *Blood* 134**,** 2036-2045.

Millot, F., Guilhot, J., Baruchel, A., Petit, A., Bertrand, Y., Mazingue, F., Lutz, P., Vérité, C., Berthou, C., Galambrun, C., Sirvent, N., Yakouben, K., Schmitt, C., Gandemer, V., Reguerre, Y., Couillault, G., Mechinaud, F., and Cayuela, J.-M. (2014). Impact of early molecular response in children with chronic myeloid leukemia treated in the French Glivec phase 4 study. *Blood* 124**,** 2408-2410.

Millot, F., Guilhot, J., Nelken, B., Leblanc, T., De Bont, E.S., Békassy, A.N., Gadner, H., Sufliarska, S., Stary, J., Gschaidmeier, H., Guilhot, F., and Suttorp, M. (2006). Imatinib mesylate is effective in children with chronic myelogenous leukemia in late chronic and advanced phase and in relapse after stem cell transplantation. *Leukemia* 20**,** 187-192.

Millot, F., Maledon, N., Guilhot, J., Güneş, A.M., Kalwak, K., and Suttorp, M. (2019). Favourable outcome of de novo advanced phases of childhood chronic myeloid leukaemia. *Eur J Cancer* 115**,** 17-23.

Muramatsu, H., Takahashi, Y., Sakaguchi, H., Shimada, A., Nishio, N., Hama, A., Doisaki, S., Yagasaki, H., Matsumoto, K., Kato, K., and Kojima, S. (2011). Excellent outcomes of children with CML treated with imatinib mesylate compared to that in pre-imatinib era. *International Journal of Hematology* 93**,** 186-191.

Suttorp, M., Schulze, P., Glauche, I., Göhring, G., Von Neuhoff, N., Metzler, M., Sedlacek, P., De Bont, E., Balduzzi, A., Lausen, B., Aleinikova, O., Sufliarska, S., Henze, G., Strauss, G., Eggert, A., Kremens, B., Groll, A.H., Berthold, F., Klein, C., Groß-Wieltsch, U., Sykora, K.W., Borkhardt, A., Kulozik, A.E., Schrappe, M., Nowasz, C., Krumbholz, M., Tauer, J.T., Claviez, A., Harbott, J., Kreipe, H.H., Schlegelberger, B., and Thiede, C. (2018). Front-line imatinib treatment in children and adolescents with chronic myeloid leukemia: results from a phase III trial. *Leukemia* 32**,** 1657-1669.

Zwaan, C.M., Rizzari, C., Mechinaud, F., Lancaster, D.L., Lehrnbecher, T., Van Der Velden, V.H., Beverloo, B.B., Den Boer, M.L., Pieters, R., Reinhardt, D., Dworzak, M., Rosenberg, J., Manos, G., Agrawal, S., Strauss, L., Baruchel, A., and Kearns, P.R. (2013). Dasatinib in children and adolescents with relapsed or refractory leukemia: results of the CA180-018 phase I dose-escalation study of the Innovative Therapies for Children with Cancer Consortium. *J Clin Oncol* 31**,** 2460-2468.
